# Wide variations in the alpha angle reporting of the hip in asymptomatic individuals—a systematic review

**DOI:** 10.1093/jhps/hnaf040

**Published:** 2025-08-04

**Authors:** Martin Jakobsen, Maria Biesèrt, Mikael Sansone, Ayeni R Olufemi, Ioannis Kostogiannis

**Affiliations:** Department of Orthopaedics, Clinical Sciences, Lund University, Skånes Universitetssjukhus, Wigerthuset, Remissgatan 4, 22185, Lund, Sweden; Department of Orthopaedics, Clinical Sciences, Lund University, Skånes Universitetssjukhus, Wigerthuset, Remissgatan 4, 22185, Lund, Sweden; Department of Orthopaedics, Sahlgrenska University Hospital, Sahlgrenska Academy, Gothenburg University, Gothenburg, Sweden; Division of Orthopaedic Surgery, McMaster University, Hamilton, Canada; Department of Orthopaedics, Clinical Sciences, Lund University, Skånes Universitetssjukhus, Wigerthuset, Remissgatan 4, 22185, Lund, Sweden

## Abstract

The alpha angle is commonly used to quantify cam-type morphologies in hips. However, the threshold values that define a pathological alpha angle remains disputed. To identify the threshold values for the alpha angle measured on X-ray, computed tomography (CT), and magnetic resonance imaging (MRI) in asymptomatic individuals in different radiological modalities. Using the Preferred Reporting Items for Systematic reviews and Meta-Analysis guidelines and checklist, a literature search was conducted in May 2024 by two independent reviewers. The PubMed, Embase, Cochrane, and CINAHL databases were searched using the following search terms: (‘Alpha angle’) AND ((‘FAI’) OR ‘femoroacetabular impingement’) AND ((((Imaging) OR CT) OR MRI) OR X ray). Of the studies identified, those measuring the alpha angle using either X-ray, CT, or MRI in an asymptomatic population were included. Study population and, if stated, demographics like gender, ethnicity, and activity level were then compiled using Excel as well as number of individuals and hips, imaging modality and view, method of evaluating symptoms, mean alpha angle, and 95% reference interval. Twenty-five articles were included in the review. Threshold values varied depending on what radiological method was used, with thresholds for X-ray ranging from 61.0° to 122.9°, thresholds for CT ranging from 50.0° to 95.3°, and thresholds for MRI ranging from 52.7° to 103.5°. According to current literature, threshold values indicating increased risk of FAI syndrome vary physiologically and depend on imaging view and modality. Therefore, no single cut-off reliably defines cam morphology and measurements should be interpreted within their clinical context.

## INTRODUCTION

Femoroacetabular impingement (FAI) syndrome is a known cause of hip and groin pain among the active adolescent population and young adults. Formally described in 1991, it is characterized by morphological variations of the hip joint causing abutment of the femoral head–neck junction and the acetabular rim [1]. The syndrome has been identified as a risk factor for developing osteoarthritis later in life, since the abnormal mechanical contact can result in repetitive microtrauma, which eventually leads to labral and chondral damage of the acetabulum [2]. With FAI syndrome increasingly being diagnosed, diagnostic criteria have evolved and created ambiguity. Therefore, the *Warwick Agreement* was established in 2016 and FAI syndrome was described as a triad of symptoms, clinical signs, and imaging findings [3].

The morphological variations associated with FAI syndrome can be divided into three subtypes: pincer, cam, and mixed. Cam morphology is defined as an osseous bump, causing a femoral head–neck asphericity [2]. To quantify the cam morphology, the most frequently used morphological parameter is the alpha angle, first described by Nötzli *et al.* [4]. Even though the alpha angle is well established clinically, no consensus has been made on the upper reference interval threshold values. The reported normal alpha angle has varied, with pathological threshold values ranging from 50 to 80° [5–8] in the literature. Furthermore, different imaging modalities and projections have been used with the aim of best quantifying the alpha angle [9–13].

Larson *et al.* [14] showed that a greater alpha angle was a predictor for hip and groin pain. However, many patients with positive radiological parameters associated with FAI syndrome do not have any symptoms. Using the current threshold values, a high prevalence of cam morphology in asymptomatic populations has been reported by several authors, concluding that these values need to be redefined to improve specificity [6, 8, 15].

Therefore, the purpose of this study was to systematically review the literature, to determine the upper reference interval threshold values for the alpha angle in asymptomatic individuals using different radiological modalities and projections. Secondarily, the aim was to investigate the impact of patient attributes such as age, gender, and ethnicity on alpha angle thresholds.

## METHODS

A systematic review was performed using the Preferred Reporting Items for Systematic reviews and Meta-Analysis (PRISMA) guidelines and using a PRISMA checklist [16]. A registration was made in PROSPERO prior to conducting the review. Registration ID CRD42021236790.

### Search strategy

In May 2024 two reviewers conducted a literature search using the following search terms: (‘Alpha angle’) AND ((‘FAI’) OR ‘femoroacetabular impingement’) AND ((((‘Imaging’) OR ‘CT’) OR ‘MRI’) OR ‘X ray’). The PubMed, Embase, Cochrane, and CINAHL databases were searched. Subsequently, studies were screened for inclusion by the reviewers independently.

### Inclusion and exclusion criteria

All studies measuring the alpha angle on either plain radiographs (X-ray), computed tomography (CT), or magnetic resonance imaging (MRI) in asymptomatic individuals were included independent of radiographic view. All non-English articles, reviews, case studies, and conference abstracts were excluded. Furthermore, studies in symptomatic patients, patients with comorbidities such as developmental dysplasia of the hip (DDH), Legg-Calvé-Perthes disease, osteoarthritis, or slipped capital femoral epiphysis were excluded. Studies examining joints other than the hip, using other imaging modalities—i.e. ultrasound, evaluating surgical outcome, being conducted exclusively on cadavers or computer models, or studies of paediatric patients, as defined by age <18, were also excluded. Lastly, due to the risk of the same asymptomatic control group occurring in multiple studies, we chose to exclude studies comparing symptomatic patients to asymptomatic controls. Disparities between the reviewers in the final selection were resolved through discussion, weighing in the opinion of a third reviewer.

### Data analysis

Study level of evidence was noted from the studies or assessed using the Oxford Centre for Evidence-Based Medicine (OCEBM) guidelines [17]. Study quality was graded using the CEBM critical appraisal of cross-sectional study tool [18].

Patient population, evaluation of symptoms and, if stated, demographics like gender, ethnicity, and activity level was then compiled using Excel 2018™ (Microsoft, Redmond, USA) as well as number of individuals and hips, imaging modality and view, mean alpha angle, and 95% reference interval (RI). In articles where no 95% RI was stated, it was calculated by adding two standard deviations (SD) to the mean. Consequently, studies stating neither the upper 95% RI of the alpha angle nor the SD of the mean were excluded, as a threshold could not be calculated. Lastly, all threshold values were rounded to one decimal place to be more easily comparable.

## RESULTS

**Figure 1:**
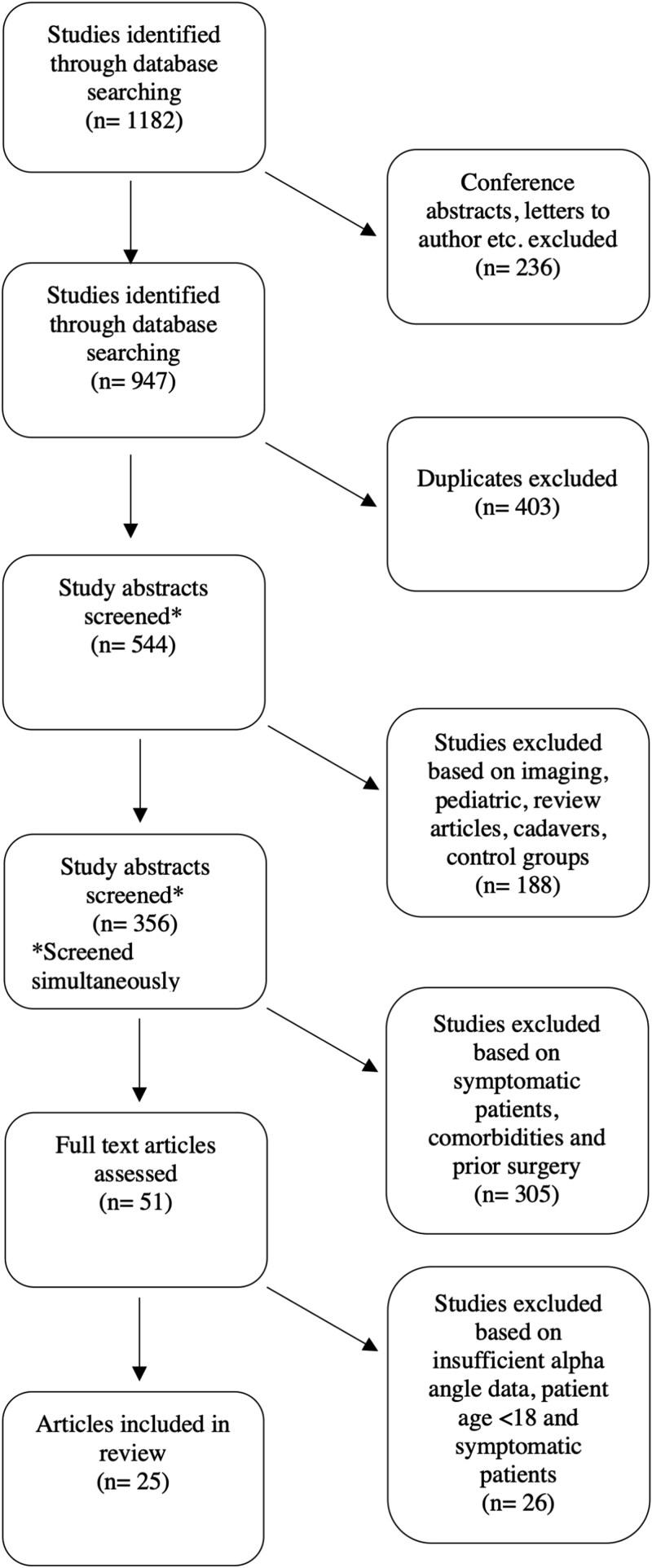
Systematic selection of studies identified from database search based on PRISMA guidelines.

From the four databases, 1182 studies were identified. Of these, 236 non-complete studies, i.e. conference abstracts or letters to authors, were excluded. Of the 947 studies remaining, duplicates accounted for 403, leaving 544 studies to be screened for inclusion. After consideration according to the inclusion and exclusion criteria, 51 studies were selected for further examination. Insufficient alpha angle data for calculation of the upper 95% RI threshold, symptomatic patients, and/or patients under the age of 18 not detected during screening left a further 26 studies being excluded. Thus, 25 studies were ultimately included. Six of these were conducted using MRI [11, 19–23], eleven were conducted using CT [5, 10, 24–32], and seven were conducted using X-ray [7, 13, 33–37]. One study was conducted using both MRI and X-ray [38]. The selection process was documented in a PRISMA flow chart (Fig. 1). Study quality was subsequently graded using the CEBM Critical appraisal of cross-sectional study tool (Table 1).

**Table 1:** Study quality graded using the CEBM critical appraisal of cross-sectional study tool.

**Study**	**AlMousa *et al.***	**Balci *et al.***	**Bento *et al.***	**Ergen *et al.***	**Falotico *et al.***	**Fraitzl *et al.***	**Gollwitzer *et al.***	**Hack *et al.***	**Han *et al.***	**Ho *et al.***	**Jung *et al.***	**Lahner *et al.***	**Lepage-Saucier *et al.***	**Malhotra *et al.***	**Mascarenhas *et al.***	**Mayes *et al.***	**Mimura *et al.***	**Mineta *et al.***	**Morales-Avalos *et al.***	**Onwuzu *et al.***	**Palmer *et al.***	**Pollard *et al.***	**Scheidt *et al.***	**Sanpatchayapong *et al.***	**Tsitskaris *et al.***
Design	PCS	PCS	PCS	PCS	PCS	RCS	RCS	PCS	RCS	PCS	RCS	PCS	PCS	RCS	PCS	PCC	RCS	RCS	RCS	RCS	PC	PCS	PCS	PCS	RCS
1. Did the study address a clearly focused question/issue?	yes	yes	yes	yes	yes	yes	yes	yes	yes	yes	yes	yes	yes	yes	yes	yes	yes	yes	yes	yes	yes	yes	yes	yes	yes
2. Is the research method (study design) appropriate for answering the research question?	yes	yes	yes	yes	yes	yes	yes	yes	yes	yes	yes	yes	yes	yes	yes	yes	yes	yes	yes	yes	yes	yes	yes	yes	yes
3. Is the method of selection of the subjects (employees, teams, divisions, organizations) clearly described?	yes	yes	yes	yes	yes	yes	yes	yes	yes	yes	yes	yes	yes	yes	yes	yes	yes	yes	yes	yes	yes	yes	yes	yes	yes
4. Could the way the sample was obtained introduce (selection) bias?	yes	yes	yes	yes	no	yes	yes	no	yes	yes	yes	yes	no	yes	no	yes	yes	yes	yes	yes	yes	no	yes	yes	yes
5. Was the sample of subject’s representative with regard to the population to which the findings will be referred?	yes	yes	yes	yes	yes	yes	yes	yes	yes	no	yes	yes	yes	yes	yes	no	yes	yes	yes	yes	yes	yes	can’t tell	yes	yes
6. Was the sample size based on pre-study considerations of statistical power?	yes	no	yes	no	no	no	no	yes	no	no	no	no	no	no	no	no	yes	yes	yes	no	no	no	no	no	no
7. Was a satisfactory response rate achieved?	yes	yes	yes	yes	yes	yes	yes	yes	yes	yes	yes	yes	yes	yes	yes	yes	yes	yes	yes	yes	yes	yes	yes	yes	yes
8. Are the measurements likely to be valid and reliable?	yes	yes	yes	yes	yes	yes	yes	yes	yes	yes	yes	yes	yes	yes	yes	yes	yes	yes	yes	yes	yes	yes	yes	yes	yes
9. Was the statistical significance assessed?	yes	yes	yes	yes	yes	yes	yes	yes	yes	yes	yes	yes	yes	yes	yes	yes	yes	yes	yes	yes	yes	yes	yes	yes	yes
10. Are confidence intervals given for the main results?	yes	yes	yes	yes	yes	yes	yes	yes	yes	yes	yes	yes	yes	yes	yes	yes	yes	yes	yes	yes	yes	yes	yes	yes	yes
11. Could there be confounding factors that haven’t been accounted for?	yes	yes	yes	yes	yes	yes	yes	no	yes	yes	yes	yes	yes	yes	yes	yes	yes	yes	yes	yes	yes	yes	yes	yes	yes
12. Can the results be applied to your organization?	yes	yes	yes	yes	yes	yes	yes	yes	yes	yes	yes	yes	yes	yes	yes	yes	yes	yes	yes	yes	yes	yes	yes	yes	yes

PCS, prospective cross-sectional study. RCS, retrospective cross-sectional study. PCC, prospective case–control study. PC, prospective cohort study.

All studies included used slightly different imaging protocols. While some variations might occur, protocols were subsequently grouped into imaging views based on similarity. In total, 13 different imaging views were used across the different modalities.

For studies examining the alpha angle using X-ray, we found estimated threshold values of 61–122.9° for the anteroposterior view, 62–84.9° for the lateral views, and 62.2° for the Dunn 45 view.

Using CT, the estimated threshold values were 61–77.8° for the three-dimensional view, 50–69° for the axial oblique view, and varying between 55.7 and 95.3° in the radial, double oblique, anteroposterior, superoinferior, and intermediate views.

**Figure 2:**
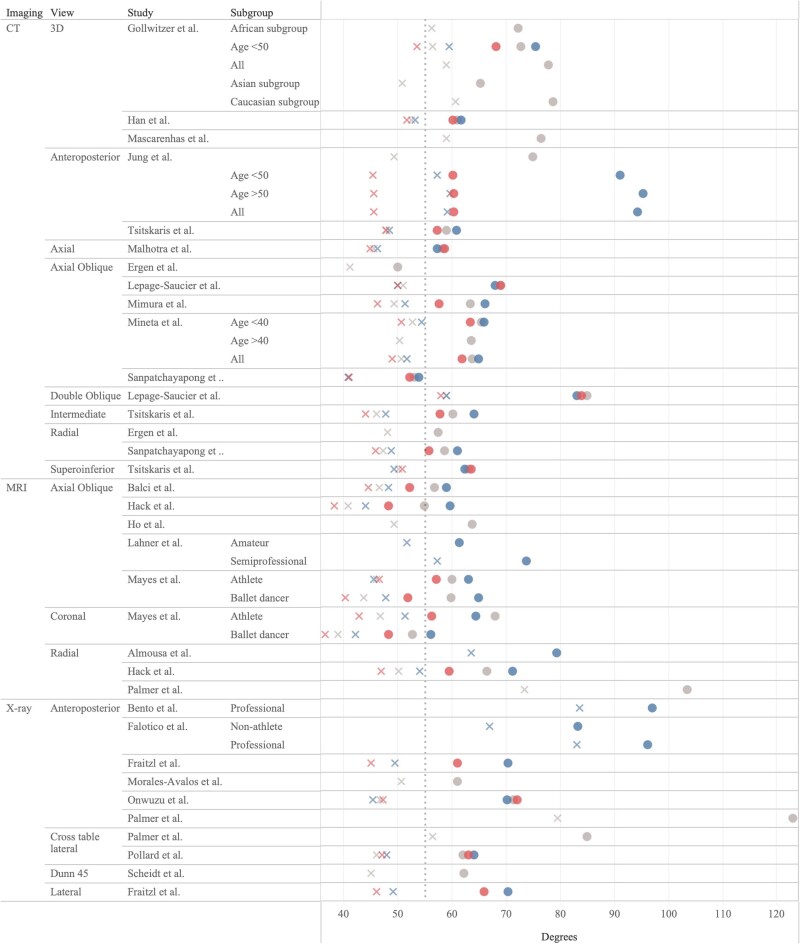
Results of all studies. Xs represents mean alpha angles and circles represent threshold value. Blue represents male, red represents female, and grey represent mixed-gender samples.

Finally, in the studies using MRI, we found estimated threshold values of 54.9–73.7° using the axial oblique view, 66.4–103.5° for the radial view, and 52.7–67.9° in the coronal view. Results of all studies are shown in Table 2 and are summarized in a more accessible manner in Fig. 2.

**Table 2:** Results from all studies.

**Study**	**Level of evidence**	**Imaging used**	**View**	**Evaluation of symptoms**	**Population type**	**Demographics**	**No. of individuals**	**No. of hips**	**Mean alpha angle (± SD)**	**95% RI threshold** ^a^
AlMousa *et al.* [23]	III	MRI	Radial view. 1:00 o’clock anterosuperior position	MRI, PROMS, clinical testing	Asymptomatic volunteers with no history of hip disease	Male. Mean age 24.2 (20–32)	48	96	63.5 (± 7.9)	79.3
Balci *et al.* [19]	IV	MRI	Axial Oblique	MRI, PROMS, clinical testing	Volunteers with no prior history of hip disease	Male (44) and Female (35). Age 18–49	79	158	46.64 (± 5.07)	56.8
						Male	44	88	48.3 (± 5.34)	59.0
						Female	35	70	44.55 (± 3.82)	52.2
Bento *et al.* [36]	III	X-ray	Anteroposterior view	X-ray, medical history, clinical testing	Professional soccer players	Male. Mean age 25,5 (18–38)	59	118	83.6 (± 6.7)	97.0
Ergen *et al.* [24]	III	CT	Axial Oblique	CT, medical history, clinical testing	Patients undergoing CT for reasons other than hip disease	Male (38) and Female (30). Age 19–46	68	131	41.1 (± 4.44)	50.0
			Radial view (anterosuperior segment A6)						48.13 (± 4.63)	57.4
Falotico *et al.* [33]	III	X-ray	Anteroposterior view	X-ray, medical consultation, clinical testing	Professional soccer players and non-athlete controls from the orthopaedic ER without hip symptoms.	Male. Professional soccer players. Age 18–40	60	120	83.0 (± 6.6)	96,2
						Male. Non-athlete controls. Age matched. Mean age 29.2	32	64	67.0 (± 8.1)	83.2
Fraitzl *et al.* [34]	III	X-ray	Anteroposterior view	X-ray	Patients suffering trauma with no signs of underlying hip pathology on X-ray	Male. Mean age 47 (SD 17)	170	170	49.4 (± 10.5)	70.4
						Female. Mean Age 55 (SD 19)	169	169	45.0 (± 8.0)	61.0
			Lateral view			Male. Mean age 47 (SD 17)	170	170	49.1 (± 10.6)	70.3
						Female. Mean Age 55 (SD 19)	169	169	46.1 (± 9.9)	65.9
Gollwitzer *et al.* [10]	III	CT	3D - Max mean AA found in the anterosuperior position (most prominently at 1:36 o’clock)	CT	Random patients with no morphological hip disease seeking medical attention for reasons unrelated to the hip	Male and female (55.2% versus 44.8% respectively). Mean age 61.2 (18–93) Ethnic information available in 89.5% of patients.	656	656	59.0 (± 9.4)	77.8
						Subgroup aged 18–50			56.4 (± 8.2)	72.8
						Male subgroup aged 18–50			59.43 (± 8.04)	75.5
						Female subgroup aged 18–50			53.45 (± 7.35)	68.2
						Caucasian subgroup	456	456	60.74 (± 8.99)	78.7
						Asian subgroup	103	103	50.76 (± 7.21)	65.2
						African subgroup	19	19	56.26 (± 7.95)	72.2
Hack *et al.* [11]	III	MRI	Axial Oblique - Anterior 3:00 o’clock position	MRI, medical history, clinical examination	Volunteers with no prior history of hip disease	Male and female (44% versus 56%), mean age 29.4 (21.4–50.6), ethnic 79% Caucasian	200	400	40.78 (± 7.05)	54.9
						Male subgroup	89	178	44.02 (± 7.82)	59.7
						Female subgroup	111	222	38.19 (± 5.06)	48.3
			Radial multiplanar - anterosuperior 1:30 o’clock			Male and female (44% versus 56%), mean age 29.4 (21.4–50.6), ethnic 79% Caucasian	200	400	50.15 (± 8.13)	66.4
						Male subgroup	89	178	54.09 (± 8.54)	71.2
						Female subgroup	111	222	46.99 (± 6.19)	59.4
Han *et al.* [25]	III	CT	3D - Max mean AA found in the anterosuperior position (most prominently at 1:00 o’clock)	CT	Patients suffering trauma with no signs of underlying hip pathology on X-ray	Korean male and female. Mean age 38.5 (± 9.2). Age range 20–54	100	100	52.45 (± 4.28)	61.0
						Male subgroup mean age 37.5 (SD 8.9). Age range 20–54	51	51	53.17 (± 4.27)	61.7
						Female subgroup mean 39.5 (SD 9.6). Age range 20–54	49	49	51.71 (± 4.21)	60.1
Ho *et al.* [20]	III	MRI	Axial Oblique	MRI, PROMS, clinical testing	Volunteers with no prior history of hip disease	Male (*n* = 10) and female (*n* = 9). Age 18–35	19	19	49.3 (± 7.20)	63.7
Jung *et al.* [26]	III	CT	Anteroposterior view	CT	Patients undergoing abdominal or pelvic CT for other medical diseases unrelated to the hip, with no morphological hip disease	Male (*n* = 108) and female (*n* = 272)	380	755	49.3 (± 12.8)	74.9
						Male subgroup mean age 62.5 (26.6–92.6)	108	215	59.12 (± 17.61)	94.3
						Male age > 50		166	59.59 (± 17.87)	95.3
						Male age < 50		49	57.21 (± 16.95)	91.1
						Female subgroup mean age 59.5 (25.5–90.9)	272	540	45.47 (± 7.4)	60.3
						Female age > 50		433	45.52 (± 7.4)	60.3
						Female age < 50		107	45.31 (± 7.46)	60.2
Lahner *et al.* [21]	IV	MRI	Axial Oblique	MRI, medical history, PROMS, Clinical testing	Semiprofessional and amateur soccer players with no prior history of hip disease	Male semiprofessional soccer players. Mean age 23.3 ± 3.3 (18–30)	22	22 Kicking leg	57.3 (± 8.2)	73.7
								22 Right leg	57.0 (± 8.3)	73.6
								22 Left leg	55.4 (± 6.5)	68.4
						Male amateur soccer players. Mean age 22.5 ± 3.5 (18–29)	22	22 Kicking leg	51.7 (± 4.8)	61.3
								22 Right leg	51.8 (± 4.8)	61.4
								22 Left leg	52.2 (± 4.8)	61.8
Lepage-Saucier *et al.* [5]	III	CT	Axial Oblique (anterior, alpha angle 90°)	CT, medical history	Asymptomatic patients with no history of hip disease undergoing CT for unrelated reasons	Male and female. Mean age 49.0 ± 16.6	94	188	51.0 (± 9.0)	69.0
						Male. Mean age 47.1 ± 16.9	49	98	50.0 (± 9.0)	68.0
						Female. Mean age 51.2 ± 16.2	45	90	50.0 (± 9.0)	69.0
			Double Oblique (anterosuperior, alpha angle 45°)			Male and female. Mean age 49.0 ± 16.6	94	188	59.0 (± 13.0)	85.0
						Male. Mean age 47.1 ± 16.9	49	98	59.0 (± 12.0)	83.0
						Female. Mean age 51.2 ± 16.2	45	90	58.0 (± 13.0)	84.0
Malhotra *et al.* [27]	III	CT	Axial	CT	Asymptomatic patients presenting with hip fracture of the contralateral hip. No prior history of hip disease	Male (*n* = 39) and female (*n* = 46). Mean age 56 (40–81)	85	85	45.63 (± 6.27)	58.2
						Male subgroup	39	39	46.31 (± 5.46)	57.2
						Female subgroup	46	46	44.93 (± 6.89)	58.7
Mascarenhas *et al.* [28]	III	CT	3D - 1:00 o’clock anterosuperior position	CT, Medical history, PROMS	Asymptomatic patients with no history of hip disease undergoing CT for unrelated reasons	Male (*n* = 49) and female (*n* = 45). Mean age 34.8 (± 7.2). Age range 18–44	94	188	58.9 (± 8.8)	76.5
Mayes *et al.* [22]	III	MRI	Axial Oblique (anterior AA)	MRI, PROMS, Clinical testing	Ballet dancers and non-ballet athletes with no history of hip disease	Professional ballet dancers. Male (*n* = 15) and female (*n* = 18). Mean age 28 (± 8). Age range 18–41. 85% Caucasian	33	66	43.6 (± 8.1)	59.8
						Male subgroup	15	30	47.7 (± 8.6)	64.9
						Female subgroup	18	36	40.2 (± 5.8)	51.8
						Age (± 2 years) and sex matched athletes. Tennis and basketball players	33	66	46.0 (± 7.0)	60.0
						Male subgroup	15	30	45.5 (± 8.8)	63.1
						Female subgroup	18	36	46.5 (± 5.3)	57.1
			Coronal (superior AA)			Professional ballet dancers. Male (*n* = 15) and female (*n* = 18). Mean age 28 (± 8). Age range 18–41. 85% Caucasian	33	66	38.9 (± 6.9)	52.7
						Male subgroup	15	30	42.1 (± 7.0)	56.1
						Female subgroup	18	36	36.6 (± 5.8)	48.2
						Age (± 2 years) and sex matched athletes. Tennis and basketball players	33	66	46.7 (± 10.6)	67.9
						Male subgroup	15	30	51.4 (± 6.5)	64.4
						Female subgroup	18	36	42.8 (± 6.7)	56.2
Mimura *et al.* [29]	III	CT	Axial oblique S5 view.	CT	Patients undergoing abdominal or pelvic CT for other medical diseases unrelated to the hip, with no morphological hip disease	Japanese Male (*n* = 57), female (*n* = 46) Mean age 54.9 (± 14.8)		103	49.2 (± 7.1)	63.4
						Male subgroup		57	51.3 (± 7.4)	66.1
						Female subgroup		46	46.2 (± 5.7)	57.6
Mineta *et al.* [30]	III	CT	Axial oblique – 2:00 o’clock anterosuperior position	CT	Patients undergoing abdominal or pelvic CT for other medical diseases unrelated to the hip, with no morphological hip disease	Japanese patients. Mean age 58.2 (± 14.8). Age range 20–89		1 178	50.6 (± 6,6)	63.8
						Male subgroup. Mean age 59.5 (± 14.4) Age range 22–89		695	51.7 (± 6.6)	64.9
						Female subgroup. Mean age 56.4 (± 15.2). Age range 20–88		483	49.0 (± 6.4)	61.8
						Young subgroup. Age < 40. Mean age 30.7 (± 9.5). Age range 20–39		154	52.6 (± 6.4)	65.4
						Male age < 40. Mean age 30.7 (± 5) Age range 22–39		78	54.4 (± 5.8)	66.0
						Female age < 40. Mean age 30.7 (± 5) Age range 20–39		76	50.6 (± 6.4)	63.4
						Elderly subgroup. Age > 40. Mean age 62.4 (± 10,8). Age range 40–89		1 024	50.3 (± 6.6)	63.5
Morales-Avalos *et al.* [35]	III	X-ray	Anteroposterior view	X-ray	Patients undergoing X-ray for evaluation of traumatic injuries No morphological hip disease	Mexican male (*n* = 640) and female (*n* = 299). Mean age 31.0 (± 9,2)	939	1878	50.6 (± 5.2)	61.0
Onwuzu *et al.* [37]	IV	X-ray	Anteroposterior view	X-ray, Medical history	Patients with no history of pelvic pathology	African male (*n* = 34) and female (*n* = 70)	104	104	46.7 (± 12.34)	71.4
						Male subgroup	34	34	45.35 (± 12.35)	70.1
						Female subgroup	70	70	47.32 (± 12.37)	72.1
Palmer *et al.* [38]	III	MRI and X-ray	Radial view. 1:00 o’clock anterosuperior position	MRI, PROMS, Clinical testing	SibKids cohort. Hereditary risk of osteoarthritis without current morphological hip disease	Male and female. Mean age 52.0. Age range 36–67	34	34	73.44 (± 15.04)	103.5
			Anteroposterior view						79.47 (± 21.72)	122.9
			Cross-table lateral view						56.39 (± 14.26)	84.9
Pollard *et al.* [7]	III	X-ray	Cross table lateral with 15° internal rotation	X-ray, Medical history, Clinical testing	Asymptomatic volunteers from the general population. Spouses or partners of patients involved in cohort studies	Male and female.	83		46–49	62.0
						Male subgroup. Mean age 47,5 (± 12). Age range 28–69	39		48.0 (± 8)	64.0
						Female subgroup. Mean age 44.4 (± 11). Age range 22–67	44		47.0 (± 8)	63.0
Scheidt *et al.* [13]	III	X-ray	Dunn view 45^°^	X-ray, Medical history, Clinical testing	Asymptomatic volunteers with no history of hip disease	Male (*n* = 28) and female (*n* = 54). Mean age 50.4, range (40–60)	82	164	45 (± 8.6)	62.2
Sanpatchayapong *et al.* [32]	IV	CT	Axial Oblique view	CT, Medical history, Clinical testing	Asymptomatic patients with no history of hip disease	Thai male (*n* = 56) and female (*n* = 61). Mean age 35.9 (18–45)	117	226	40.9 (± 6.06)	53.0
						Male subgroup	56	109	40.82 (± 6.55)	53.9
						Female subgroup	61	117	40.98 (± 5.58)	52.1
			Radial view – 1:30 o’clock anterosuperior position			Thai male (*n* = 56) and female (*n* = 61). Mean age 35.9 (18–45)	117	226	47.27 (± 5.66)	58.6
						Male subgroup	56	109	48.71 (± 6.12)	61.0
						Female subgroup	61	117	45.93 (± 4.86)	55.7
Tsitskaris *et al.* [31]	III	CT	Superoinferior view	CT, PROMS	Patients ≤40 years of age undergoing abdominal and pelvic CT for abdominal trauma or non-specific abdominal pain	Male (*n* = 21) and female (*n* = 24). Mean age 33, range (20–40)	45		50.1 (± 6.5)	63.1
						Male subgroup	21		49.2 (±6.6)	62.4
						Female subgroup	24		50.8 (± 6.4)	63.6
			Intermediate view			Male (*n* = 21) and female (*n* = 24). Mean age 33, range (20–40)	45		46.1 (± 7)	60.1
						Male subgroup	21		47.8 (±8.1)	64.0
						Female subgroup	24		44.0 (±6.9)	57.8
			Anteroposterior view			Male (*n* = 21) and female (*n* = 24). Mean age 33, range (20–40)	45		48.0 (± 5.5)	59.0
						Male subgroup	21		48.4 (± 6.2)	60.8
						Female subgroup	24		47.7 (±4.8)	57.3

^a^Mean alpha angle +2 × SD or as stated in the article. SD, standard deviation. RI, reference interval.

## DISCUSSION

The main findings of this systematic review are that the alpha angle varies widely depending on the imaging view and radiologic modality utilized. In general, the results indicate that the currently used alpha angle threshold values of 55–60° may be a low threshold considering what is normal in an asymptomatic population. This further emphasizes that the diagnosis of FAI syndrome cannot be made on morphology alone and that clinical examination and symptoms must be considered. More details regarding the specific differences in imaging modality and projections, as well as individual study limitations, are described in Appendix S1.

Nötzli *et al.* [4] described in 2002 in a study of 39 patients with clinical signs of FAI syndrome, a threshold value of 50° for both genders using MRI. These values have later been adapted to CT and X-ray, and a common threshold of 55° has been used [15]. Some studies have reported a higher prevalence of increased alpha angle, using higher cut-off values [11]. Sutter *et al.* [8] proposed an increase of threshold value in MRI from 55 to 60° to increase specificity. Nevertheless, it is important to note that increasing the threshold value could potentially increase the specificity, but increasing it too much would lower the sensitivity.

van Klij *et al.* [39] conducted a systematic review in 2020 with the aim of investigating different alpha angle threshold values for defining cam morphology. A threshold value > 60° was presented as the most appropriate cut-off value. In comparison, our systematic review focuses solely on asymptomatic individuals to determine thresholds for physiological alpha angles. Furthermore, we compared different radiological modalities and views since imaging differences might lead to threshold value variations. Patient attributes such as age, gender, activity level, and ethnicity were also noted and briefly discussed.

The definition of asymptomatic varied across articles, with some relying on medical history alone and others using a combination of medical history, patient-reported outcome measures (PROMS), and clinical examination. The presence of cam morphology alone does not fully explain symptom development, with other factors including acetabular anatomy, version, and spinopelvic kinematics also contributing. Furthermore, patient engagement in provocative activities may affect symptom manifestations.

Which imaging modality and view that best visualizes a cam morphology is subject to debate [40]. While CT and MRI often are considered the gold standard as they provide more information in three dimensions than X-ray, they are also more costly and less available for screening purposes [7, 40]. Furthermore, Nepple *et al.* [40] found a sensitivity of 86% to 90% when comparing CT to the Dunn 45°, anteroposterior, cross table lateral, and frog leg views on X-rays, suggesting that a series combining X-ray views can be used to accurately diagnose cam morphologies.

Three studies examined the correlation between alpha angle and age [10, 26, 30]. Jung *et al.* [26] found no significant age-related differences in alpha angle values, while Mineta *et al.* [30] observed significant differences between young and elderly groups in the axial oblique view, using age 40 as the cutoff. Gollwitzer *et al.* [10] also found significant age-related differences but considered them clinically irrelevant, attributing them to early degenerative changes in osteoarthritis.

Regarding gender differences, results were more diverging. Statistically significant gender differences were found in six studies [10, 11, 26, 29, 30, 32] while not being found in five studies [5, 7, 22, 27, 37]. Typically, gender differences have been explained by differences in the natural development of the femoral head–neck region as well as bone adaptations to excessive physical activity in males, especially during the male growth spurt in adolescence [41, 42]. Others have also suggested that they might arise from subclinical epiphyseal slip, which is more common in males [43]. However, why gender differences are only observed by some studies remains unclear.

Whilst most of the included studies in this review mainly examined western populations, some also studied people of other ethnicities, including Asian [10, 25, 32], Indian [27], and African populations [37]. In general, these studies found a lower prevalence of FAI syndrome and a lower estimated alpha angle threshold compared to those found in studies of Caucasian populations. This aligns with the fact that most cases of osteoarthritis in Asians are secondary to other pathologic processes such as developmental dysplasia, idiopathic osteonecrosis, rheumatoid arthritis, Legg-Calvé-Perthes disease, or post-infectious arthritis [44]. Consistent with other studies [6, 41, 42], Falotico *et al.* [33], Lahner *et al.* [21], Bento *et al.* [36], and Mayes *et al.* [22] demonstrate that activity level and type of activity also affects the alpha angle, as people who are athletically active tend to have higher alpha angles than those who are not.

As demonstrated in the previous section, numerous variables such as gender, ethnicity, and activity level might affect the alpha angles in a certain population and hence, affect the RI for what is considered normal in an asymptomatic population. It is, however, important to note that while the RIs between populations can differ, this might not necessarily translate into them needing different cut-off values in a diagnostic setting. While all included populations in this study are in some way considered asymptomatic, the differences in RIs might correlate with the risk of developing symptoms of FAI syndrome at a later stage.

Some studies, including van Klij *et al.* [39], argue that 95% RIs might be an incorrect way of diagnosing cam morphology because of its high prevalence in asymptomatic individuals. However, the findings of this review suggest that the previously used cut-off values of 50–60° might instead be too low, resulting in an over-estimation of population prevalence [4, 7]. Furthermore, the difficulties in the distinction of symptomatic and asymptomatic hips on radiographic findings alone emphasize the importance of clinical examination and level of symptoms as stated in the *Warwick agreement*.

The limitations of the current review include the inclusion of adult patients over 18, thus limiting generalizability to paediatric populations. Also, due to the relatively small number of studies, especially for some radiological views, no meta-analysis of the data could be performed. Therefore, our results are presented in a narrative fashion. Finally, there was a predominance of North American and European studies, making the findings less robust for other populations. To improve diagnostic accuracy in the future, further studies examining more complex imaging tools, spinopelvic kinematics, and the introduction of artificial intelligence are needed.

In conclusion, according to the current literature, there is a wide physiological variation in the threshold values considered to increase the risk of FAI syndrome. This variation is furthermore dependent on which imaging views and radiologic modalities are used. Consequently, a single universal threshold angle cannot reliably determine the presence of cam morphology, and measurements should instead be interpreted within a wider clinical context.

## Supplementary Material

APPENDIX_1_hnaf040

## Data Availability

This review article does not include any new experimental data. All data analysed and discussed are from previously published studies, which are cited within the article. The original sources of the data can be accessed through the respective publications.

## LEVEL OF EVIDENCE

IV

## TRIAL REGISTRATION

The review was registered in PROSPERO prior to conducting the search. Registration ID CRD42021236790.
