# Supplementary material for: Wide variations in the alpha angle reporting of the hip in asymptomatic individuals—a systematic review
Source: J Hip Preserv Surg. 2025 Aug 4;13(1):45–55. doi: 10.1093/jhps/hnaf040 (PMC12891998; doi:10.1093/jhps/hnaf040)
Supplement: APPENDIX_1_hnaf040 [file appendix_1_hnaf040.docx]

**APPENDIX 1**

**X-ray**

Of the 25 studies included in this review, eight examined patients using X-ray imaging techniques (7,13,33-38). The two main views being used were the anteroposterior view and lateral views, while the Dunn 45 view was only used in a single study (13).

In the six studies examining patients using anteroposterior views, estimated 95% threshold values varied between 61° to 122.9° (33-38). Arguably, the outlier in this group is the study by Palmer et al. (38), with an estimated threshold value of 122.9. This difference can probably be attributed to several reasons. Although the study, unlike the studies by Fraitzl et al. (34) and Morales-Avalos et al. (35) that had threshold values in the 60-70° range, clinically tested patients for symptoms associated with FAI syndrome, only 34 patients were included. This increases the risk of having a handful of extremes in the study sample and skew the results in either direction compared to the population in general. Also, although patients in the Palmer et al. (38) study were only included if they had not had investigation or treatment for hip pain in the previous two years, they partly consisted of people from the SibKids cohort - being family members of patients that have had total hip arthroplasty following end stage osteoarthritis. Based on the clinical assessment, the hip with the greatest suspicion of FAI syndrome was then examined. These factors indicate that, while the study by Palmer et al. (38) does not specifically fulfil any of our exclusion criteria, the estimated threshold values might be less suitable for general application. Interestingly, in a study of 120 asymptomatic hips in professional soccer players and 64 hips in non-athlete controls Falotico et al. (33) found threshold values of 96.2° and 83.2° respectively. Both of these groups were tested clinically and should thus be less likely to include individuals that are not truly asymptomatic. Similarly, the study by Bento et al. (36) included 56 asymptomatic professional soccer players that, after clinical examination, presented a threshold value of 97°.

Lateral views were utilized by three studies (7,34,38). In the study by Fraitzl et al. (34), threshold values can be estimated to 70.3° and 65.9° for men and women respectively. This compares to the study by Pollard et al. (7) that, although using a smaller patient population, found threshold values of 62-64° in 83 men and women that were clinically tested for symptoms of FAI syndrome. The estimated threshold value from the study by Palmer et al. (38) was again higher at 84.9°.

Lastly, the Dunn 45 view was only tested by Scheidt et al. (13) in a study of 164 hips from a total of 82 individuals that underwent clinical examination. While the estimated threshold value of 62.2° resembles what is found in other views, the question arises whether or not both hips in a single individual can truly be considered independent observations.

**CT**

In the three studies (10,25,28) using a 3D approach measuring the alpha angle, all identified the largest alpha angle in the anterosuperior segment of the femoral head. 853 hips all together were examined and the threshold values varied between 61 ° to 77.8 °. Mascharenas et al. (28) and Gollwitzer et al. (10) had large sample sizes and similar threshold values of 76.5 ° and 77.8°. Gollwitzer et al. (10) examined different ethnicity subgroups and found a similar threshold value of 65.2° among their Asian subgroup, which is similar to the Korean patient group of 100 hips studied by Han et al. (25) with a threshold value of 61°. Gollwitzer et al. (10) concluded that alpha angle values varied significantly between the Asian subgroup and the Caucasian subgroup, which had a threshold value of 78.7° (P < 0.0005).

It is important to note that, while none of the patients included in the study by Gollwitzer et al. (10) had morphological signs of hip disease and all sought medical attention for other reasons than hip problems, they were never asked specifically whether they had any hip symptoms or pain. That means that some of the patients included in the material potentially could have symptoms if clinically tested and therefore values might be lower if those patients were excluded. Gollwitzer et al. (10) also had an African subgroup, but since it only consisted of 19 patients, they deemed the sample too small to make assumptions about a population of African ethnicity. Interestingly, no gender differences were present in either of the studies.

In the axial oblique view both Mimura et al. (29) and Mineta et al. (30) studied Japanese hips, while Sanpatchayapong et al. (32) studied a thai population. Mimura et al. (29) found a threshold value among 103 hips of 63.4°. Mineta et al. (30) included 1178 hips and presented a threshold alpha angle value of 63.8°. These are similar results as presented in the Asian population in the 3D view by both Han et al. (25) and Gollwitzer et al. (10). Sanpatchayapong et al. (32) however, found a little lower threshold value of 53° in their study of 226 hips. Overall, the threshold values vary a lot in the axial oblique view. Ergen et al. (24) found a threshold value of 50°, Lepage-Saucier et al. (5) found a value of 69°, while Malhotra et al. (27) had a value in between using an axial view. The disparity could potentially be caused by different factors, like lack of standardized measurement and differences in patient gender distribution, ethnicity and activity level.

A few of the studies also present less common projections using the CT.

Ergen et al. (24) and Sanpatchayapong et al. (32) both also evaluated the alpha angle in the radial projection presenting a thresholds values of 57.4° and 58.6° respectively, while Lepage-Saucier et al. (5) used the double oblique view and found the threshold value of 85°. Both of these CT projections can roughly be compared to the 3D view since both visualize the anterosuperior region of the femoral neck. Both Tsitkaris et al. (31) and Jung et al. (26) used CT views that are not as common in the literature. Although similar threshold values are presented further studies are needed for comparison and confirmation of these values obtained by these methods.

**MRI**

Of the four studies measuring the alpha angle in the axial oblique view the results suggest a range of threshold values between 54.9 ° to 73.7 °. Balci et al. (19) and Hack et al. (11) present similar results in both the mixed group 56.6 ° and 54.9 °, as well as men vs female. Ho et al. (20) with a lot smaller cohort presented a higher threshold value of 63.7 °. Lahner et al. (21) indicated that individuals that are very athletically active might have a higher alpha angle compared to amateur athletes. Hack et al. (11), Almousa et al. (23) and Palmer et al. (38) all evaluated radial view. Hack et al. (11) measured the alpha angle in the anterosuperior 1:30 o’clock position, examining 400 hips, and presented a threshold value of 66.4 °. This is rather similar to other previous studies. Almousa et al. (23) examined 96 hips in 48 male volunteers and presented a threshold value of 79.3° in the anteriosuperior 1 o’clock position. Although somewhat higher, the threshold value presented by Almousa et al. (23) is similar to the one presented by Hack et al. (11) in their male subgroup (71.2°). With an estimated threshold value of 103.5° in the anteriosuperior position, the study by Palmer et al. (38) is yet again an outlier as described above. Lastly Mayes et al. (22) who compared different athletes in the coronal view presented lower alpha angle values among ballet dancers at 52.7 ° compared to basketball and tennis players at 67.9 °.
